# The Title of Doctor and What It Now Signifies in Our Own and Other Countries

**Published:** 1907-06

**Authors:** Arthur G. Hobbs

**Affiliations:** Atlanta, Ga.


### *THE TITLE OF DOCTOR AND WHAT IT NOW SIGNIFIES IN OUR OWN AND OTHER COUNTRIES.*

By Arthur G. Hobbs, M.D., Atlanta, Ga.

What is the significance of this title gradually coming to be, if indeed it has not already reached a climax? This sentence may at first appear ambiguous to any man who is called doctor (and his name is legion without regard to his previous condition or servitude), but when he stops to think he will, no doubt, appreciate its significance. By whatever route he may have gained the title he will see it, or even if he has never earned it he may feel it. It would be hazardous to risk a guess at the proportions of those who have earned it, to those who have assumed it, and to those who have had it thrust upon them.

The appellation of doctor is now as generally and almost as gratuitously given, in our Southland especially, as is that of Captain or Major or Colonel, by the stranger on the streets, by the news-boy, by the cab driver, by the street-car conductor or, by the Pullman porter. Any man is likely to be so addressed if he looks like a gentleman in their eyes, whether he may or may not wear Vandyke whiskers. And yet if he should wear a goatee and a soft hat, they would probably address him as Captain or Colonel.

But who can blame this class of the laity for applying the title so indiscriminately, when we have no other laity than the army officers, the attorneys-at-law, the railroad officials and the drummers! The druggists and the chemists and the dentists are doctors; and woe be to him who is having his corns trimmed if he does not call his chiropodist, doctor!

Then what are we to do with the divinity doctors—the D.D.'s? Are they not doctors too? But how are we, of the M.D. persuasion, supposed to know when we should say Mr. or when we should say Dr. to our own minister? He might take umbrage at us, in case he had just been notified by a special delivery letter, that the title of

D.D. had been conferred upon him, when we knew nothing of the momentous event.

And so it is with the L.L.D.'s and the D.C.L.'s. Even Mr. Roosevelt has recently had this evidence of his erudition, or of his strenuosity, conferred upon himself by one or more of the leading Universities. ( However, not many of us will, in this case, gainsay the justice, if indeed the conferred title of doctor still carries with it any evidence of knowledge and distinction. ) Our President could demand, if he chose, that he be called Dr. Roosevelt and not Mr. President. Edward Guelph, of England, also has had these honors both from Oxford and Cambridge. So he, too, could demand that he be called Dr. Edward Guelph instead of Edward VII of England, if he chose. Then we must now assume that all statesmen of renown and all politicians of notoriety, have already had the degrees conferred upon themselves by some neighboring University, so it would rarely be a technical mistake to address any of this class as "doctor."

When I once addressed a noted ophthalmlc ourgeod—Mr. Prichett of London—as doctor, he replied to me, "Please, Dr., address me as Mr. Pritchett, or without the Mr. if you like." I hesitated a moment in deference to his polite rebuke, when he added, "Dr., I must address you so, as an American physician and surgeon, but here in London we have gotton over all that." Then he said, "As it would make but little difference and add no distinction, I have not gone to the trouble of signing the papers that would make me an M.D. and have myself called Dr. instead of Mr."

There are some who call themselves doctors—and it is only natural that others should follow their suggestion and so address them —whose sole business in life is to pander to the superstitions of our colored sisters, that their twisted locks can be changed into curly hair. The doctor furnishes the bright colored fluid to their pastors, the dollars flow in by a multiple of the rule of three, then two of them are at once addressed as "doctor."

The role of the druggist-doctor is sometimes similar to this. If the victim is able to get to the druggists, she asks him for some medicine for her floating misery, her colic, her bronchitis, or for her consumption. The druggist-doctor has it already prepared, directions and all, in a bright red bottle. But if she sends to him because she can't walk when she has typhoid fever, pneumonia, tonsilar abscess or anything else but appendicitis, she gets the same colored fluid, with the same directions and from the same "doctor." The ignorance and the cupidity of the one sometimes seems to work harmoniously with the knavery or ignorance of the other, and vice versa, and thus there is an endless chain.

The patient (the sufferer) has misunderstood what the title of doctor should really mean, until after she has spent all of her money, or possibly until she is about to go where all good sufferers go, before she has learned that she might have applied at first to a Dr. to get a respite from her floating miseries, at least for a term of years before her departure hence.

One may not be called an iconoclast who only asks what we, as M.D.'s should do under these circumstances, even if he does not suggest something better. Should we assume some other and more distinctive title, or should we fiight it out on our original rights that have come down to us from mediaeval times? Or should we do as our friend, the attorney-at-law does and use the Mr. followed by physician and surgeon? Our M.D. title has been changed to meet the emergencies by the statesmen, the politicians, the college presidents, the dentists, the ministers, the veterinaries and the druggists, into L.L.D., D.C.L., D.D.S., D.D., D.V.S., Ph.D., while the patent medicine man, the optician, the chiropodist and the head musician have assumed the title outright. It would appear then that we have no laymen, except the Army and Navy officers, (and not all of them), our friends, the attorneys-at-law, the railroad officials, the commercial travelers, the cops and dagos.

805-807 and 809 English-American Bldg.

---

The Medical Era of St. Louis, has absorbed the Medical Mirror, hitherto published in the same city. This consolidation is in harmony with the spirit of the day, a reduction in the number of journals and adding strength to the survivors. We wish many years of prosperity and influence to The Medical Era.

